# Design and Implementation of a Randomized Controlled Trial of Genomic Counseling for Patients with Chronic Disease 

**DOI:** 10.3390/jpm4010001

**Published:** 2014-01-08

**Authors:** Kevin Sweet, Erynn S. Gordon, Amy C. Sturm, Tara J. Schmidlen, Kandamurugu Manickam, Amanda Ewart Toland, Margaret A. Keller, Catharine B. Stack, J. Felipe García-España, Mark Bellafante, Neeraj Tayal, Peter Embi, Philip Binkley, Ray E. Hershberger, Wolfgang Sadee, Michael Christman, Clay Marsh

**Affiliations:** 1 Division of Human Genetics, Ohio State University Wexner Medical Center, Columbus, OH 43420, USA; E-Mails: Amy.Sturm@osumc.edu (A.C.S.); Kandamurugu.Manickam@osumc.edu (K.M.); Amanda.Toland@osumc.edu (A.E.T.); Ray.Hershberger@osumc.edu (R.E.H.); 2 Center for Personalized Health Care, Ohio State University Wexner Medical Center, Columbus, OH 43420, USA; E-Mail: Clay.Marsh@osumc.edu; 3 Coriell Institute for Medical Research, 403 Haddon Avenue, Camden, NJ 08103, USA; E-Mails: erynn.gordon@invitae.com (E.S.G.); TSchmidl@coriell.org (T.J.S.); margaret.keller@redcross.org (M.A.K.); cstack@acponline.org (C.B.S.); fgarciaespana@coriell.org (J.F.G.-E.); mbellafante@coriell.org (M.B.); christman@coriell.org (M.C.); 4 Invitae, 458 Brannan Street, San Francisco, CA 94107, USA; 5 American Red Cross, 700 Spring Garden Street, Philadelphia, PA 19123, USA; 6 American College of Physicians, 190 N. Independence Mall West, Philadelphia, PA 19106, USA; 7 Department of Internal Medicine, Ohio State University College of Medicine, Columbus, OH 43420, USA; E-Mail: Neeraj.Tayal@osumc.edu; 8 Department of Bioinformatics, Ohio State University College of Medicine, Columbus, OH 43420, USA; E-Mail: Peter.Embi@osumc.edu; 9 Division of Cardiovascular Medicine, Ohio State University College of Medicine, Columbus, OH 43420, USA; E-Mail: Philip.Binkley@osumc.edu; 10 Program in Pharmacogenomics, Department of Pharmacology, The Ohio State University College of Medicine, Columbus, OH 43420, USA; E-Mail: Wolfgang.Sadee@osumc.edu

**Keywords:** implementation, genomics, medicine, randomized, patients, counseling, actionable, risk perception, pharmacogenomics

## Abstract

We describe the development and implementation of a randomized controlled trial to investigate the impact of genomic counseling on a cohort of patients with heart failure (HF) or hypertension (HTN), managed at a large academic medical center, the Ohio State University Wexner Medical Center (OSUWMC). Our study is built upon the existing Coriell Personalized Medicine Collaborative (CPMC^®^). OSUWMC patient participants with chronic disease (CD) receive eight actionable complex disease and one pharmacogenomic test report through the CPMC^®^ web portal. Participants are randomized to either the in-person post-test genomic counseling—active arm, *versus* web-based only return of results—control arm. Study-specific surveys measure: (1) change in risk perception; (2) knowledge retention; (3) perceived personal control; (4) health behavior change; and, for the active arm (5), overall satisfaction with genomic counseling. This ongoing partnership has spurred creation of both infrastructure and procedures necessary for the implementation of genomics and genomic counseling in clinical care and clinical research. This included creation of a comprehensive informed consent document and processes for prospective return of actionable results for multiple complex diseases and pharmacogenomics (PGx) through a web portal, and integration of genomic data files and clinical decision support into an EPIC-based electronic medical record. We present this partnership, the infrastructure, genomic counseling approach, and the challenges that arose in the design and conduct of this ongoing trial to inform subsequent collaborative efforts and best genomic counseling practices.

## 1. Introduction

Genomic technologies are increasingly being utilized in the clinical setting and are expected to transform personalized approaches to medicine over the next decade. When genomic biomarkers are coupled with clinical information, family history, lifestyle, and other environmental factors, more informed predictions about risks for rare and common disease and response to therapeutics can be provided [1]. In addition, genomic information can be used to tailor screening and prevention strategies. While personalized medicine is still in the early stages of development, a number of academic medical centers and integrated health systems have already begun to use discrete genomic data for personalized clinical care [2,3,4]. However, the clinical implementation of large amounts of genomic information is emerging [5,6], and studies examining the effects of genomic counseling on this process have primarily focused on healthy motivated cohorts [1,7,8,9]. Hence, it is imperative to evaluate diverse populations of patients with chronic disease (CD), their understanding and response to actionable genomic risk information, related to and beyond their diagnosis, and to develop optimal methods for information delivery [10,11,12,13].

The Coriell Personalized Medicine Collaborative (CPMC^®^) is an ongoing longitudinal study, launched in December 2007, by the Coriell Institute for Medical Research to explore the core issues of utility and delivery of personalized medicine [14]. The Ohio State University Wexner Medical Center (OSUWMC) partnered with Coriell in 2009 to focus on the use and delivery of personalized medicine to a CD population. Specifically, this collaboration centered on recruitment of a new cohort of the CPMC^®^ to investigate the impact of genomic counseling using a randomized controlled trial study design in patients with CD disease within an academic medical center environment. Patients with either a diagnosis of heart failure (HF) or hypertension (HTN) were selected because of high disease prevalence, frequent clinic visits, and long-term use of medications for disease management. All prospective CD participants were required to, (1) attend a one-hour informed consent and study education session, and (2) register on the CPMC^®^ web portal through their home computer and complete CPMC^®^ parent study surveys (demographics, medical history, family history, lifestyle, medications, medication reactions) and a baseline study specific survey. Genotyping was then performed. All participants were subsequently notified by email of the availability of an initial batch of results for eight health conditions (type 1 diabetes, type 2 diabetes, coronary artery disease, age-related macular degeneration, prostate cancer, melanoma, systemic lupus erythematous, and hemochromatosis) and one pharmacogenomic result (*CYP2C19* results interpreted in the context of response to clopidogrel) through the CPMC^®^ web portal. To determine the impact of genomic counseling on outcomes, participants were block randomized to either receive in-person genomic counseling (active arm) or no genomic counseling (control arm). Active participants were offered in-person genomic counseling after viewing of at least one test report through the CPMC^®^ web portal. The control arm was offered in-person genomic counseling after a three-month randomization period. Follow up OSUWMC-Coriell study specific surveys are then administered to both groups through the CPMC^®^ web portal. Additional health condition and pharmacogenomic (PGx) reports are released over the course of study, along with additional parent CPMC^®^ study surveys.

The primary study outcome is to determine whether there is a change in risk perception and understanding of test results for participants who receive in-person genomic counseling (active arm) *versus* those that will not (control arm) at baseline compared to follow-up. Study specific baseline and follow-up surveys measure risk perception, risk perception accuracy, general and relative risk numeracy, genetic knowledge, intention to change health behavior, acceptability of test result information, and other outcome measures related to the participant’s risk for the initial batch of results. We will measure satisfaction with genomic counseling and modifications to health behaviors over time. OSUWMC physicians were also recruited into a pilot study to explore their knowledge and test result utilization. Here we report on clinical implementation and study activities over the 28-month period, from 30 May 2011 to 31 September 2013. The study continues to collect follow-up data.

## 2. Methods

The parent CPMC^®^ study was established as a prospective observational research study with ancillary randomized controlled trials seeking to determine the impact of multiplex genomic test results on health and health behavior when reported directly to study participants. It has also sought to understand best practices around the use of genomic information in medical settings and individual health management within three study cohorts: community, cancer, and CD. The community cohort participants were recruited without regard to health status or affiliation with a healthcare institution, while the cancer study group consists of participants with a personal history of either breast or prostate cancer. The CPMC^®^ has been described in detail previously [14].

The OSUWMC-Coriell partnership and this study focused on the CD cohort, and the randomization of this cohort to assess the impact of genomic counseling on various study outcomes. To develop the necessary study related infrastructure, the educational pieces and infrastructure currently in place as part of the CPMC^®^ parent study were adapted and transformed. This included customization of the CPMC^®^ web portal to incorporate study-specific surveys, allowing access to study participant reports by the OSUWMC licensed genomic counselors, and randomization mechanisms. An encryption system allows for transfer of the DNA sample, the CPMC^®^ test reports and phenotype/genotype data between institutions.

### 2.1. Participants

HF and HTN were chosen given the burden of these diseases and their prevalence (2.1% and 29.1% [15,16] (age-adjusted prevalence among U.S. adults aged 20 and over in 2010 and adults 18 and over in 2011–2012, respectively). Direct involvement of OSUWMC physicians was achieved through the identification of clinical champions to assist with patient recruitment. OSUWMC patient participants are required to be age 18 or older, have access to the Internet, and a diagnosis of either HF or HTN since the implementation of their EPIC^®^ electronic medical record (EMR) system (06/2008).

### 2.2. Study Procedures

**Figure 1:**
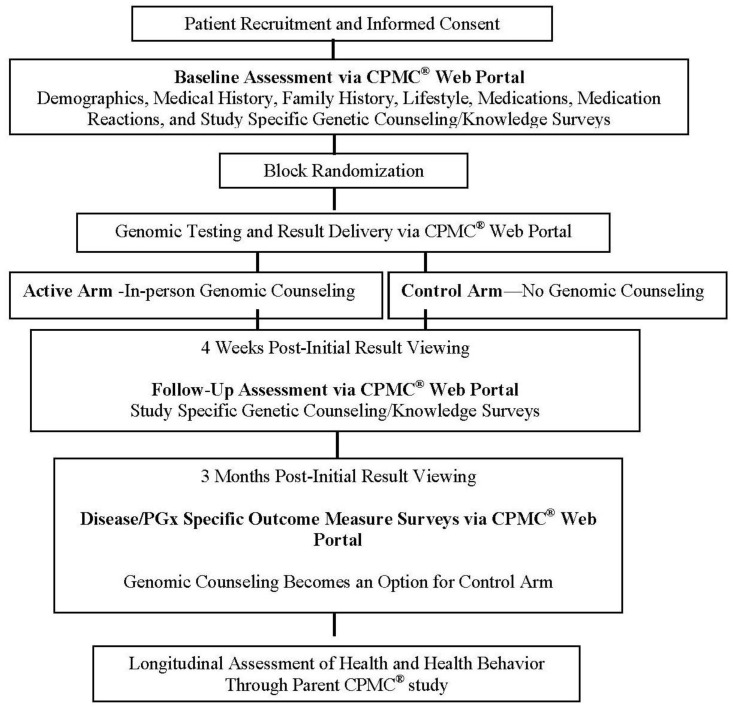
Study schema.

Patients were enrolled in the clinical setting by a trained study recruiter who administered a PowerPoint educational presentation on all aspects of the study including access to the CPMC^®^ web portal, the randomization component, background information on DNA, genes, and single nucleotide polymorphisms, composition of the test reports, and the availability of genomic counseling. The participant then completed the informed consent process and a saliva sample was collected for DNA testing. Samples and consent documentation were sent to Coriell, and samples were accessioned and unique CPMC^®^ web portal accounts created for each participant. All participants completed baseline on-line surveys (demographic, medical history, family history, lifestyle, medications, and study-specific) using the CPMC^®^ web portal on their home computer. Online support was available if a participant had questions regarding access of the surveys through the CPMC^®^ web portal, otherwise survey completion was unsupervised. Participants, however, will be prompted to review and update the data entered into the web portal periodically to provide longitudinal health data from which changes in health outcomes will be determined as part of the parent CPMC^®^ study. Following completion of all baseline surveys, participants were block randomized to the active or control arm of the study. Each arm received the same nine personalized risk reports for their initial batch of test reports. Participants in the active arm received in-person genomic counseling, from one of two available licensed genomic counselors, within one month of viewing at least one report of the initial batch of nine reports. In contrast, subjects in the control arm were not offered genomic counseling as part of the study protocol, but were able to access in-person genomic counseling, if requested, three-months post-result viewing and post-follow up survey (study) completion (Figure 1).

### 2.3. Variants Returned

Genetic testing with Affymetrix 6.0 and DMETPlus GeneChips was performed only after participants completed the required baseline questionnaires. These GeneChip platforms were chosen to allow study of behavioral outcomes related to diseases affecting a reasonable proportion of all CPMC^®^ study participants. Results reported to participants were limited to single nucleotide polymorphism (SNP) variants associated with complex diseases and drug-gene pairs deemed potentially actionable by Coriell’s advisory boards—the Informed Cohort Oversight Board (ICOB) and the Pharmacogenomic Advisory Group (PAG) [14]. A potentially actionable condition is a condition for which the risk is likely to be mitigated by individual action (behavior or lifestyle) or by medical action (screening, preventive treatment or early intervention). The initial batch of results for the chronic disease cohort includes eight actionable diseases and one drug-gene pair (Table 1).

**Table 1:** Initial results: health conditions and relative risk based on SNP variant result.

Health Condition	SNP	Reference Genotype (RR)	Heterozygote (RR)	Homozygote (RR)
Type 1 Diabetes	rs9272346	GG (0.08)	GA (0.3)	AA (1.0)
Type 2 Diabetes	rs7754840	GG (1.0)	GC (1.2)	CC (1.3)
Hemochromatosis	rs1800562	GG (1.0)	AG (1.0) *	AA **
Systemic Lupus Erythematosus	rs3821236	GG (1.0)	AG (1.4)	AA (2.0)
Coronary Artery Disease	rs1333049	GG (1.0)	GC (1.3)	CC (1.7)
Prostate Cancer ***	rs16901979	CC (1.0)	CA (1.5)	AA (1.5)
Skin Melanoma	rs910873	CC (1.0)	CT (1.7)	TT (3.0)
Age Related Macular Degeneration	rs10490924	GG (1.0)	GT (2.4)	TT (6.0)

RR is relative risk; * Males Only, lifetime risk 4%; ** Males with 2 copies of the risk variant (AA) are 27 times as likely to develop hemochromatosis RR = 27 (lifetime risk 57%). Females with GG or GA result have no relative risk provided, but are told they have up to a 1% lifetime risk. Females with AA result have no relative risk provided but are told they have a 16% lifetime risk of developing hemochromatosis; *** Both male and female participants receive result report, with risk only applicable to male participants.

These eight health conditions were chosen in accordance to the Coriell Institute guiding principles for risk reporting: relative high frequency of the genetic variant used to assess risk; varied effect size of each variant on risk; and the finding that each condition has an actionable or potentially actionable component [17]. Most of these conditions are also highly prevalent in the general population. The *CYP2C19* report was the first drug-gene pair to be released through the CPMC^®^. The development of CPMC^®^ risk reports has been described in detail previously [17]. Following the completion of the follow up assessment (post-genomic counseling for active arm and post-result viewing for control arm), additional approved personalized risk (Table 2) and drug response reports (Table 3) are prospectively released to OSUWMC-Coriell CD participants through the CPMC^®^ web portal. This will allow for longitudinal assessment of health and health behavior through the parent CPMC^®^ study.

### 2.4. Post-Test Genomic Counseling

**Figure 2:**
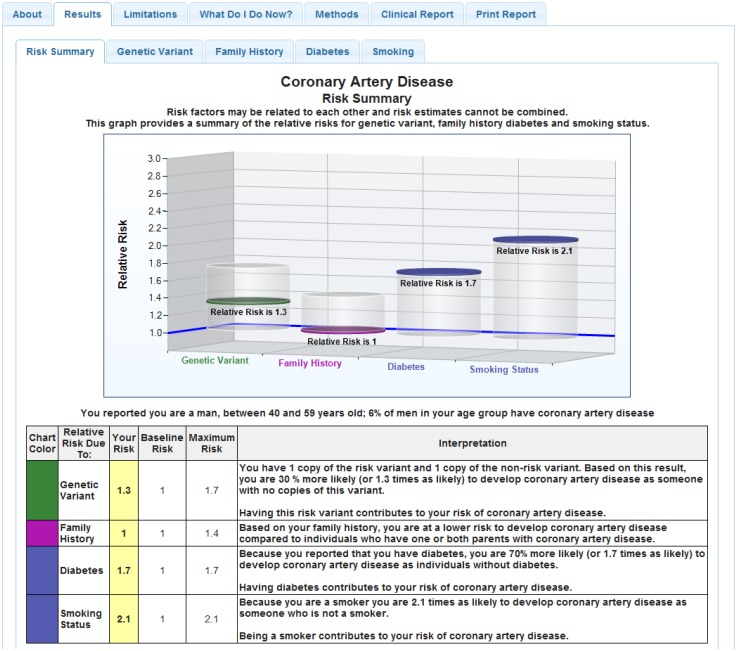
Sample CPMC^®^ coronary artery disease report.

**Figure 3:**
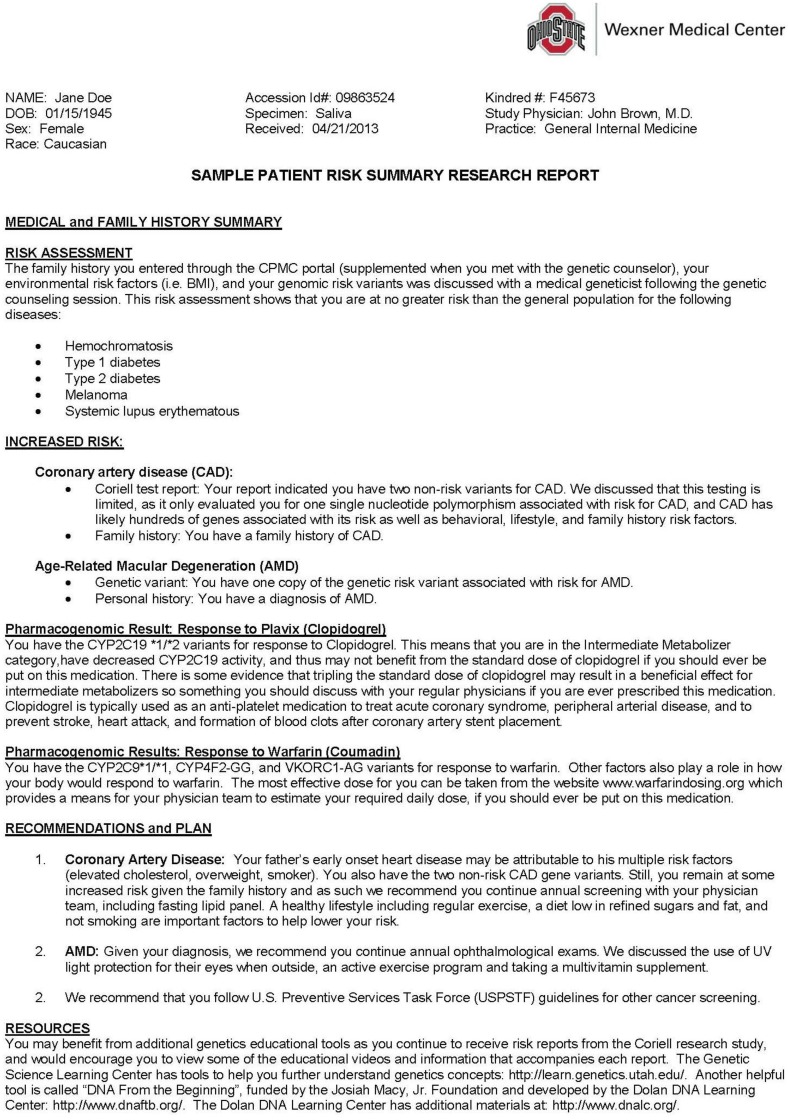
Sample patient risk summary research report.

Genetic counseling protocols for Mendelian disorders as well as those available in the context of multiplex genomic studies were reviewed and content areas catalogued to develop the design of the genomic counseling session [7,18,19,20,21,22,23,24,25,26]. The one-hour, in-person genomic counseling sessions provide individualized risk assessment using the nine personalized CPMC^®^ study reports, collection of at least a three-generation pedigree, as well as evaluation of the patient’s medical history, social history, pertinent environmental risk information, and current health promotion and screening behaviors. Prior to the genomic counseling session, the patient’s medical history is extracted from the OSUWMC EMR, and the family history entered into the CPMC^®^ web portal by the patient is extracted to develop a pedigree. As the genomic counseling sessions were designed to review results for all nine personalized reports, these are also accessed. This individual patient information is then assessed by the OSUWMC genomic counselor in conjunction with the OSUWMC medical geneticist. The design of the parent study CPMC^®^ test reports (Figure 2) present risk as relative risk for each condition, for genetic and non-genetic (lifestyle; environmental) risk factors in both graphical and numeric form [17]. These reports also provide estimates of risk in the general population as a reference point. To aid explanation and contrast, and given the amount of information that must be presented in the one-hour counseling session, we developed a tabular visual display for all eight disease reports and the *CYP2C19* report. The tabular display synthesizes each of these factors into a one-page document to provide an overall “quick reference” summary of risk, and comparison of the patient’s risk to the general population. At least one CPMC report is also assessed “live” via the web portal during the session to associate with the tabular display. There is active discussion as to the essential risk factors each patient has for a given disease, to include additional disease risks that may have been identified by additional review of the family history. There is discussion to gauge what actions the patient might take to prevent or lower risk for development of a given disease. Post-session, the details of the risk assessment and the updated medical and family history is reviewed in conjunction with the medical geneticist. A risk summary research report (Figure 3), which provides focused interpretation for each of the nine personalized CPMC^®^ study reports, as well as recommendations based on this assessment and the medical and family history, is then generated. This risk summary report is mailed to the patient participant, and made available to the OSUWMC health care team through the EMR. 

**Table 2:** Additional Coriell Personalized Medicine Collaborative (CPMC^®^) approved health conditions for prospective release.

Health Condition
Breast Cancer Colorectal Cancer Ulcerative Colitis Crohn’s Disease Obesity Rheumatoid Arthritis Testicular Cancer Chronic Obstructive Pulmonary Disease Bladder Cancer Celiac Disease Lung Cancer Osteoporosis Asthma Osteoarthritis Multiple Sclerosis Intracranial Aneurysm Exocrine Pancreatic Cancer Ischemic Stroke

**Table 3:** Additional CPMC^®^ approved drug-gene pairs for prospective release.

Drug-Gene Pairs
CYP2C19/PPIs SLCO1B1/Simvastatin CYP2C9/Celecoxib TPMT/Thiopurines CYP2C9/Warfarin VKORC1/Warfarin CYP4F2/Warfarin CYP2D6/Codeine ATM/Metformin IL28A, IL28B, ITPA and Interferon/Ribavirin CYP2D6&CYP2C19/Amitriptyline/Nortriptyline CYP2D6/Paroxetin

### 2.5. Study-Specific Surveys

Study-specific baseline and follow up surveys were designed to measure perceived personal risk, risk perception accuracy, general and relative risk numeracy, genetics/genomics knowledge, intention to change health behavior, personalized medicine perceptions and genomics awareness, information-seeking preferences and behaviors, acceptability of test result information, family communication, perceived utility, and genomic counseling satisfaction (if randomized to that arm) [1,18,20,27,28,29,30,31,32] (Supplementary Files 1 and 2). Additional optional disease specific outcome surveys are sent to participants through the web portal three months after they view each result to measure result recall, sharing of results with family and health care providers, behavioral and lifestyle changes, and physician recommendations [14]. We can also monitor the OSUWMC EMR to determine potential outcomes on various measures and medical recommendations to elucidate changes in actual behavior during the course of study on this patient-based population.

### 2.6. Clinical Implementation (Physicians and the EMR)

OSUWMC physicians were recruited into a pilot study of their knowledge and result utilization. Two physician champions, one each from Cardiovascular Medicine (CVM), and General Internal Medicine (GIM) were identified, and internal group meetings with the respective physican teams arranged. The investigators designed an educational module consisting of an in-person lecture on genetics, genomics, pharmacogenomics, case examples, and study details including the randomization component, the makeup and availability of the CPMC^®^ test reports in the EMR, and a question and answer session. In addition, the physicians were informed of the availability of the study genomic counselors and medical geneticists for consult regarding their patients’ CPMC^®^ test reports. Interested physicians completed an informed consent document, a study survey adapted from Roederer and colleagues [33] related to their awareness of genomic medicine and perception of clinical utility, and worked with study staff to recruit eligible patients.

Clinical implementation of the OSUWMC EPIC-based EMR system occurred during the development of this study that necessitated the development of new processes. Each CPMC^®^ report was labeled with a naming standard with separate creation of a new procedure code for direct uploading to the EMR. The reports are accessible by any OSUWMC physician through hyperlinks to the report content via the EPIC/Labs tab. Patient participants seen for genomic counseling subsequently receive a risk summary research report (Figure 3), which was also routed directly to their physician participant through the EMR system. Non-participant physicians are also able to access the patient risk summary research report, and the CPMC^®^ test reports in the EMR.

### 2.7. Approach to Statistical Analysis

The statistical analysis will proceed in several distinct phases, from generating descriptive summaries, graphical representations to identify outliers, to performing inferential analyses. Survey questions will be categorized by using summary indexes such as the genetic knowledge score, the relative risk numeracy index, and the benefits of the OSUMC-CPMC study score. In addition, factor analytic techniques will be used to categorize outcomes. Descriptive statistics, such as means, standard deviations, medians and range for continuous variables (e.g., age, length of time to receive genomic counseling, length of time between baseline and follow-up surveys, numbers of risk reports viewed by the participant, genetic knowledge score), and percentages for categorical variables (e.g., gender, race, education, income) will be generated for quantitative data. These statistics will be generated for all quantitative data on the CD cohort, by genomic counseling group (active *versus* control), disease group (HF *versus* HTN) and relevant subgroups of interest. Histograms and box plots will be the graphical measures used to complement descriptive analyses of categorical data. Relevant continuous measures will be evaluated for normality and those differing markedly from normality will be summarized by using medians, interquartile ranges, and box-plots. In addition, response variables that violate assumptions of normality will be transformed to achieve normality or analyzed using nonparametric statistics. For statistical inference, we will employ a variety of parametric and non-parametric statistical techniques such as t-test, chi-square, Fisher’s exact test, Wilcoxon rank sum test, Person-r, Spearman-rho or point- biserial correlations, linear and non-linear regression analysis, random effects models, generalized estimating equations, factor analysis, and structural equation modeling. For analyses involving small sample sizes, exact statistical tests and bootstrap methods will be applied.

Overall differences in risk perception and other outcome measures between the active and the control groups will be assessed. The analyses will adjust for relevant covariates such as CD group (HF *versus* HTN), sex, race, education, income, occupation, number of risk reports viewed, genomic counselor, and others found to differ significantly between groups (*i.e.*, confound) when comparing treatment conditions (active *versus* control) on baseline characteristics. We will consider analyses based upon (1) all randomized subjects (commonly called the intention-to-treat population); and (2) excluding those who were non-compliant. The statistical tests will be two-sided with a type-I error of 0.05.

In addition, exploratory analyses to evaluate the effect of counseling by genetic and non-genetic risk (high *versus* low) interaction will be evaluated. Such interactions will help us understand if between-counseling group differences in outcome measures vary by genetic risk group. Exploratory analyses will also be performed to describe the baseline knowledge of study physicians regarding genomic testing for common complex disease and the clinical utility and integration of genomic information into electronic medical records during the course of the study.

A total of 252 patients (126 patients each in the active and control arms), provides 80% power to detect differences in changes in overall risk perception of 0.5 points (on a 5 point scale) and greater. For example, if the change in overall risk perception is 0 (no change) in the group without genomic counseling (control arm), then we would be able to detect as significantly different, changes in the counseling group (active arm) of −0.5 or smaller, and of +0.5 or greater. These estimates assume a standard deviation for change score of 1.5 (based upon internal preliminary data for three-month overall perceived risk of type 2 diabetes) and two-sided tests with α = 0.05.

## 3. Results

### 3.1. Patient Recruitment/Counseling

Of 252 patient participants accrued as of October of 2013, 210 have completed the required baseline questionnaires (Table 4). Forty-two of the patients provided consent but failed to complete the required baseline surveys, and, thus, were removed from study. All 210 have received their initial batch of nine personalized CPMC^®^ test reports, with equal randomization into the active (105) and control (105) arms. In the active group, 73 out of 105 participants have received genomic counseling. Nine out of 105 have received in-person genomic counseling in the control group.

**Table 4:** Cohort characteristics (n = 210).

Characteristic	N
**Age (years)**	58.1
**Gender**	
Male	117
Female	93
**Race**	
Caucasian	187
African American	16
Native American	1
Mixed	5
Do not want to answer	1
**Education**	
<High School	3
High school	17
Vocational school	1
Some college	45
Associate degree	26
Bachelor degree	50
Graduate degree	68

### 3.2. Physician Recruitment

Twenty General Internal Medicine (GIM) and Cardiovascular Medicine (CVM) physicians participated in the pilot study (57%; 12/27 GIM; 8/8 CVM), completing pre-post education genetics knowledge assessment, attending an in-person educational module, and working with study staff to recruit patients into the study. The fifteen GIM physicians who declined participation cited lack of time, concerns with having to act on the results for their patients (*i.e.*, screen for coronary artery disease if the patient participant had a SNP risk variant), and perceived lack of clinical utility after the initial educational intervention. As the study design required a sufficient number of physicians to be involved in order to accrue patients, a third clinical area, the Department of Family Medicine (FM) was added in October 2012. An alternate approach was developed for patient recruitment utilizing an internal FM recruitment team. Interested FM physicians could become “physician participants”, as with the GIM/CVM group, but instead of an in-person educational module, a one-hour educational webinar accredited by OSUWMC for a maximum of 1.5 AMA PRA Category 1 Continuing Medical Education Credit(s)^TM^ was made available. Although 16 FM physicians subsequently provided access to patients for recruitment, none completed the educational program or pre-post education knowledge assessment.

## 4. Discussion

In this report we detail the design and implementation of a prospective randomized controlled trial to investigate the impact of in-person genomic counseling in the return of actionable genomic information for complex disease and pharmacogenomics. Specifically, patients with chronic disease managed in an academic medical center received genomic results through the CPMC^®^-Coriell web-portal, and were randomized to additional in-person post-test genomic counseling *versus* only the web-based return of results. This academic institution-private non-profit partnership is among the first coordinated efforts to implement genomic information, including pharmacogenomic information, into patient care to permit such a study to be undertaken.

The strengths of an academic medical center include access to patients and their clinical data, robust EMR and clinical support systems, clinician scientists, expertise with human genetics, and insightful pharmacy and pharmacogenomics support. In our view these strengths outweigh the limitations and challenges of the academic medical center environment, which include institutional inertia, fragmented expertise and the lack of appreciation and acceptance by clinicians to enact genomic guided medicine [6,9,13,18]. These challenges were met with dedicated involvement of senior leadership, active engagement of physician champions and other vested parties (*i.e.*, pharmacogenomics), and integration of genetic counselors and geneticists to establish the protocol, and processes for data return and infrastructure development. The existing CPMC^®^ infrastructure provided key strengths, including experience with genomic based protocols and consent processes, CLIA-compliant genotyping, internal oversight boards to define actionable conditions, and an interactive web portal for direct delivery of participant results. Internal pilot funding from both organizations catalyzed project initiation. Through internal meetings, teleconferences and other interactions including travel to the partner institute, the partnership has evolved into a coordinated effort that takes advantage of the diversity and strengths of each institution. Limitations, such as privacy concerns for sharing medical records and genomic data, and the incorporation of outside genomic test reports into the EMR, were overcome by active involvement of interdisciplinary IT workgroups and legal teams. Significant infrastructure was developed that allows confidential sharing of data between institutions, specifically, direct release of the initial study results for uploading to the EMR (and prospectively for new test reports); extraction of clinical data from the EMR; and transfer of the genomic datasets. Lastly, modification of an existing comprehensive consent document addressed many of the confidentiality concerns present in prospective genomic-based research.

Patient participants consented to genomic testing with the understanding that the GeneChips utilized test for over one million single SNP sites of variation, but that the study results would only be released for variants associated with actionable health conditions approved by the ICOB and PAG [14]. This concept of consenting to limited and yet unknown information has not been routinely used outside of the CPMC^®^ and raised unique issues related to participant expectations [34]. However, participants were informed that they will have access to all approved CPMC^®^ results, and the option to view or not view each result independently. In addition, participants have been empowered, as part of the consent process, to indicate or deny their consent for sharing of data (genomic/phenotypic) with for-profit and non-profit third party researchers. The consent document, thus, provides what is now referred to as tiered-layered-staged consent [35]. Tiered consent allows study participants choices about the future use of their specimen beyond the initial study, as is often practiced in biobank research [22]. Layered consent involves providing only information (results) pertinent to the particular study, and providing this to all study participants; new results are then made available (layered) for access by the individual. Staged consent involves the element of time, which in this study was provided at two separate points: baseline (for enrollment purposes), and when the participant opts to view specific test reports via the CPMC^®^ web portal (both from the initial batch of results, and prospectively). Patient participants also signed a separate, subject information sheet which allowed investigators access to discrete phenotypic information extracted from the EMR and for that information as well as genomic data to be shared between institutions.

Integral to the clinical genomic translation process is effective use of EMR applications to assist the physician team at the point of care. This component involved construction of IT applications for an existing EPIC-based EMR. Test results were transmitted from Coriell to OSUWMC in PDF format, in a secure manner, labeled with the appropriate disease/PGx name (*i.e.*, Coriell Report—Plavix) and made available to the OSUWMC physician team in the EMR. However, the length and format of the CPMC^®^ test reports did not lend themselves well to rapid interpretation or automated clinical decision support platforms [36]. To address this concern, study design modification supported the use of the genomic variant information as a foundation for development of EMR-based clinical decision support (CDS) platforms [37,38]. Currently under development, these applications are based on discrete data points (*i.e.*, SNPs). They will incorporate information from the patient’s EMR such as medications combined with the genomic variant data to trigger “best practice alerts” defined via conventional logical assertions. The first CDS algorithm under development is that for *CYP2C19* variants, and will include, over time, additional CDS capabilities, based on the potential adverse effect, such as recommending against the use of a certain medication [38].

Given our interest in evaluating physician use of genomic results, OSUWMC physicians were recruited into a pilot study of knowledge and result utilization. Many of the GIM and CVM physicians who chose study participation have become active partners, with studies looking at result utilization for patient care underway. We believe the low response rate for study participation on the part of other physicians, such as those in the FM group, can be interpreted in multiple ways. Lack of time, as suggested by the GIM physicians who also declined involvement; lack of interest in genetics; lack of interest in research; or concern about data that could be gleaned from participation (e.g., lack of genetic knowledge; clinical utility). More research is needed on barriers to physician participation in a study like this, especially since emerging technologies will allow more individuals access to their genomic profiles and primary care physicians, in particular, will become more involved in communicating risk information [1,8,12,13,35,39].

Genomic tests that analyze large sets of markers across the genome for multiple diseases and PGx variants continue to proliferate [1,5,6,9]. Although these offerings may vary, many of the disease susceptibilities conveyed are for common and complex disease, and for which the genetic contribution may explain only part of the disease burden. It is speculated that being told about genetic risk of complex disease might cause individuals to overestimate the probability of developing disease. This might generate unnecessary worry, anxiety, risk, and expense [29,40], although some studies suggest otherwise [1,8,41,42]. To mitigate these concerns, genomic counseling can be employed to adequately and effectively interpret and communicate the benefits, risks, and limitations [43], and assist with interpretation and education. Although preliminary data suggest that the uptake of genomic counseling is low (10%–15%) [7,22,42] understanding participant needs may help increase utilization [12]. Whether individuals who receive genomic information act on their results also requires further exploration. This is particularly true in diverse patient-based populations who have, to date, limited access or uptake of genomic testing for complex disease, and are likely to be different in many ways from the healthy, well-educated “early adopters” studied thus far [1,8,10]. In this study, patient participants receive education on the genotyping and SNP results and provide informed consent prior to testing, with extensive educational information (text; video) for each disease report made available through the CPMC^®^ web portal. The randomization component, therefore, allows opportunity to study the utility of post-test in-person genomic counseling *versus* only the web-based return of results; to test our hypothesis of a significantly greater change in risk perception and understanding of test results for participants who receive in-person genomic counseling (active arm) as compared to those who do not (control arm). Our approach to development of the genomic counseling protocol was multifaceted, using literature review and feedback from the CPMC^®^ genomic counselors, who have had extensive experience providing phone genomic counseling to CPMC^®^ community participants. Indeed, rather than only focusing on the disease or PGx reports of interest to the participant, the in-person sessions were designed to review results for all nine personalized reports. To aid explanation, and given the amount of information discussed, we developed and incorporated a tabular visual display for all eight disease reports and the *CYP2C19* report into these patient sessions, with added discussion of variants associated with an increased risk (risk variants), decreased risk (protective variants), or those with no clinical impact. The tabular display used in the in-person counseling session synthesizes each of the risk factors into a one-page document to provide an overall “quick reference” summary. There is additional counseling for the *CYP2C19* result, which includes education regarding their specific haplotype (e.g., CYP2C19*1/*3 or *2/*2), metabolizer type, and an explanation of the indications for prescription of the relevant medication(s) (e.g., clopidogrel) [44]. The CPMC^®^ risk reports are also accessed live via the web portal during sessions to assist participants in understanding how to navigate their results moving forward. This also presents an opportunity for “teachable moments” to enforce key concepts (*i.e.*, heritability, importance of modifiable risk factors). How patients with chronic disease cognitively engage and react to this information; their perceived susceptibility and methods of coping given they already have a chronic condition; their approach to decision-making and on how best to utilize information for which they may have no prior experience; and what they may want and need from the process of genomic counseling are areas of address. As there is need for more scalable and streamlined delivery models for common complex disease, this collaborative effort allows for prospective comparison of the CD and community CPMC^®^ cohorts, and to study the effects of genomic counseling on risk perception, knowledge retention and behavior change. This may also allow results from this controlled-trial to be explored for generalizability to other populations. Additional identification of risk and subsequent referral for appropriate medical services (*i.e.*, nutritional counseling), and follow through on EMR referrals is being closely monitored, to determine whether any new referrals are dependent upon the genomic counseling assessment *versus* initiated independently by the patient’s physician.

## 5. Conclusions

Genetic counseling, like other medical professions must continue to evolve and adapt to ever increasing amounts of data, and effectively translate this data to the lay person. This randomized controlled trial is among the first coordinated efforts to implement genomic information, including pharmacogenomic information, into patient care, and to investigate the impact of in-person genomic counseling in the return of actionable genomic results for common complex disease. This study will provide critical insights into the impact of genomic counseling on behavior and health, and enable the design of larger trials. It will allow for preliminary practice recommendations regarding best practices to facilitate comprehension of genomic test results, accurate risk perception, and to motivate behavior change. It will also promote further development of genomic counseling models and delivery methods. Patient response to pharmacogenomic testing, the myriad issues surrounding physician education and integrating effective clinical decision support to providers through the EMR are also areas of future research interest.

## Supplementary Files

Supplementary File 1 Supplementary Information (PDF, 940 KB)
