# Supplementary material for: Design and Implementation of a Randomized Controlled Trial of Genomic Counseling for Patients with Chronic Disease
Source: J Pers Med. 2014 Jan 8;4(1):1–19. doi: 10.3390/jpm4010001 (PMC4051230; doi:10.3390/jpm4010001)
Supplement: Supplementary File 1 — Supplementary Information (PDF, 940 KB) [file jpm-04-00001-s001.pdf]

**Supplemental File 1. Baseline Survey Instrument**

## Perceived Likelihood of Disease

1. On a scale of 1 to 5, where 1 is certain NOT to happen and 5 is certain TO happen, what do you think is your chance of developing each of the following diseases in your lifetime?

| | **Certain NOT to Happen 1** | **2** | **3** | **4** | **Certain TO Happen 5** | **Not applicable** | **Do not want to answer** |
|---|---|---|---|---|---|---|---|
| Coronary artery disease | | | | | | | |
| Type 2 diabetes | | | | | | | |
| Iron Overload/ Hemochromatosis | | | | | | | |
| Prostate cancer | | | | | | For female participants | |
| Melanoma | | | | | | | |
| Age-related macular degeneration | | | | | | | |
| Lupus | | | | | | | |
| Type 1 diabetes | | | | | | | |
| Colorectal cancer | | | | | | | |
| Inflammatory bowel disease | | | | | | | |
| Bladder cancer | | | | | | | |
| Breast cancer | | | | | | | |
| Obesity | | | | | | | |
| Rheumatoid arthritis | | | | | | | |
| Testicular cancer | | | | | | For female participants | |
| Chronic obstructive pulmonary disease (COPD) | | | | | | | |
| Crohn's Disease | | | | | | | |
| Ulcerative colitis | | | | | | | |

## Risk Perception Accuracy

1. Do you have an increased risk for any of the following conditions due to your *family history*?

| | **Yes** | **No** | **Don't know** | **Not applicable** | **Do not want to answer** |
|---|---|---|---|---|---|
| Coronary artery disease | | | | | |
| Type 2 diabetes | | | | | |
| Iron Overload/ Hemochromatosis | | | | | |
| Prostate cancer | | | | For female participants | |
| Melanoma | | | | | |
| Age-related macular degeneration | | | | | |
| Lupus | | | | | |
| Type 1 diabetes | | | | | |
| Colorectal cancer | | | | | |
| Inflammatory bowel disease | | | | | |

2. Do you have an increased risk for any of the following conditions due to your *environmental risk* (for example, smoking, poor diet, high Body Mass Index (BMI))?

| | **Yes** | **No** | **Don't know** | **Not applicable** | **Do not want to answer** |
|---|---|---|---|---|---|
| Coronary artery disease | | | | | |
| Type 2 diabetes | | | | | |
| Iron Overload/ Hemochromatosis | | | | | |
| Prostate cancer | | | | For female participants | |
| Melanoma | | | | | |
| Age-related macular degeneration | | | | | |
| Lupus | | | | | |
| Type 1 diabetes | | | | | |
| Colorectal cancer | | | | | |
| Inflammatory bowel disease | | | | | |

3. On a scale from 1 to 5, where 1 is Not at all and 5 is Completely, how much do you think having a *genetic risk variant* determines whether or not a person will develop each of the following conditions?

| | **1 = Not at all** | **2** | **3** | **4** | **5 = Completely** | **Do not want to answer** |
|---|---|---|---|---|---|---|
| Coronary artery disease | | | | | | |
| Type 2 diabetes | | | | | | |
| Iron Overload/ Hemochromatosis | | | | | | |
| Prostate cancer | | | | | | |
| Melanoma | | | | | | |
| Age-related macular degeneration | | | | | | |
| Lupus | | | | | | |
| Type 1 diabetes | | | | | | |
| Colorectal cancer | | | | | | |
| Inflammatory bowel disease | | | | | | |

4. On a scale from 1 to 5, where 1 is Not at all and 5 is Completely, how much do you think *family history* determines whether or not a person will develop each of the following conditions?

| | **1 = Not at all** | **2** | **3** | **4** | **5 = Completely** | **Do not want to answer** |
|---|---|---|---|---|---|---|
| Coronary artery disease | | | | | | |
| Type 2 diabetes | | | | | | |
| Iron Overload/ Hemochromatosis | | | | | | |
| Prostate cancer | | | | | | |
| Melanoma | | | | | | |
| Age-related macular degeneration | | | | | | |
| Lupus | | | | | | |
| Type 1 diabetes | | | | | | |
| Colorectal cancer | | | | | | |
| Inflammatory bowel disease | | | | | | |

5. On a scale from 1 to 5, where 1 is Not at all and 5 is Completely, how much do you think *environmental risk factors* (for example, smoking, poor diet, high Body Mass Index (BMI)) determine whether or not a person will develop each of the following conditions?

| | **1 = Not at all** | **2** | **3** | **4** | **5 = Completely** | **Do not want to answer** |
|---|---|---|---|---|---|---|
| Coronary artery disease | | | | | | |
| Type 2 diabetes | | | | | | |
| Iron Overload/ Hemochromatosis | | | | | | |
| Prostate cancer | | | | | | |
| Melanoma | | | | | | |
| Age-related macular degeneration | | | | | | |
| Lupus | | | | | | |
| Type 1 diabetes | | | | | | |
| Colorectal cancer | | | | | | |
| Inflammatory bowel disease | | | | | | |

**General, Expanded, and Relative Risk Numeracy:**

*Expanded Numeracy Scale—EASY LEVEL QUESTION*

1. If the chance of getting a disease is 10%, how many people would be expected to get the disease:
A. Out of 100?
B. Out of 1000?

*General Numeracy Scale—MODERATE LEVEL QUESTION*

1. Imagine that we rolled a fair, six-sided die 1,000 times. Out of 1,000 rolls, how many times do you think the die would come up even (2, 4, or 6)?

*General Numeracy Scale—HARD LEVEL QUESTION*

1. In the ACME PUBLISHING SWEEPSTAKES, the chance of winning a car is 1 in 1,000. What percent of tickets to ACME PUBLISHING SWEEPSTAKES win a car?

*Relative Risk Numeracy Items*

1. People without a family history of coronary artery disease have a 20% risk to develop coronary artery disease. People with a family history have a relative risk of 2.0 (they are 2 times as likely to develop coronary artery disease as those without a family history). What is the risk for someone with a family history?
A. 40%
B. 20%
C. 22%
D. Don’t know
E. Do not want to answer

2. If a person has a genetic variant that gives a relative risk for developing type 2 diabetes of 1.3, how likely are they to develop type 2 diabetes compared to someone with no copies of that genetic variant?
A. 1.3 times more likely
B. 30% more likely
C. 13 times more likely
D. Both A and B
E. None of the above
F. Don’t know
G. Do not want to answer

**Genetic Knowledge Assessment:**

1. How much time did you spend on the Coriell Personalized Medicine Collaborative (CPMC) website reading about the study or about personalized medicine and genomics?
    a. Less than 5 min
    b. Between 5 min and 30 min
    c. Between 30 min and 1 h
    d. More than 1 h
2. Have you been exposed to genetics before enrolling in the CPMC (college-level courses, genetic or personalized medicine websites, articles, or books)?
    a. Yes
    b. No
    c. Don't Know
    d. Don't Want to Answer
3. Have you ever received genetic counseling?
    a. Yes
    b. No
    c. Don't Know
    d. Don't Want to Answer
4. Compared to most people, how would you rate your knowledge of genetics?
    a. Better than most people
    b. About average
    c. Less than most people
5. When you have a health problem, where do you go to get information?
    a. Your doctor
    b. The internet
    c. Library
    d. Other ______________________

    If you answered Other in the question above, where do you get information about a health problem?
6. It is possible to see a gene with the naked eye.
    a. True
    b. False
    c. Don't Know
7. Healthy parents can have a child with a hereditary disease.
    a. True
    b. False
    c. Don't Know

8. The onset of certain diseases is due to genes, environment, and lifestyle.
    a. True
    b. False
    c. Don’t Know
9. The carrier of a disease gene may be completely healthy.
    a. True
    b. False
    c. Don’t Know
10. All serious diseases are hereditary.
    a. True
    b. False
    c. Don’t Know
11. Genes are inside cells.
    a. True
    b. False
    c. Don’t Know
12. The child of a disease gene carrier is always also a carrier of the same disease.
    a. True
    b. False
    c. Don’t Know
13. A gene is a piece of DNA.
    a. True
    b. False
    c. Don’t Know
14. A gene is a part of a chromosome.
    a. True
    b. False
    c. Don’t Know
15. All body parts have all of the same genes.
    a. True
    b. False
    c. Don’t Know
16. It has been estimated that a person has about 20,000 genes.
    a. True
    b. False
    c. Don’t Know

17. A person's race and ethnicity can affect how likely they are to get a disease.
    a. True
    b. False
    c. Not sure
18. Each of us has variations in our genes that make it more likely that we will get certain diseases.
    a. True
    b. False
    c. Not sure
19. A "complex disease" is a health condition brought on by many genes and lifestyle and environment.
    a. True
    b. False
    c. Not sure
20. A single nucleotide polymorphism or "SNiP" is a variation present in some individuals that stretches across a large section of DNA.
    a. True
    b. False
    c. Not sure

Based on what you know about genetics, please indicate whether you think the following statements are true or false.

| | **True** | **False** |
|---|---|---|
| Once a genetic marker for a disorder is found in a person the disorder can be prevented or cured. | | |
| If a person has a genetic marker for a disorder, the person will always get the disorder. | | |
| Only mothers can pass on genetic disorders. | | |
| People who have a genetic marker for a disease are unhealthy. | | |
| A person's health habits can influence whether or not their genes cause disease. | | |

**Intention to Change Health Behavior:**

1. On a scale from 1 to 5, where 1 is strongly disagree and 5 is strongly agree, how much do you agree or disagree with the following statements?

| | **Strongly disagree 1** | **Disagree 2** | **Neutral 3** | **Agree 4** | **Strongly agree 5** | **Do not want to answer** |
|---|---|---|---|---|---|---|
| If my OSUMC-CPMC results tell me I have an increased risk for a condition such as coronary artery disease, I will adopt healthier habits, such as getting regular exercise, making changes to my diet, and/or stop smoking. | | | | | | |
| I intend to tell my family members about my OSUMC-CPMC results. | | | | | | |
| I intend to share my OSUMC-CPMC results with my health care provider(s). | | | | | | |

**Personalized Medicine Perceptions and Genomics Awareness:**

1. What do you think the benefits of the OSUMC-CPMC study are? Please state how strongly you agree or disagree with each.

| | **Strongly disagree** | **Disagree** | **Neither** | **Agree** | **Strongly agree** | **Do not want to answer** |
|---|---|---|---|---|---|---|
| The results will tell me what my genetic future is. | | | | | | |
| The results will tell me about all diseases I am at risk for. | | | | | | |
| The results will tell me about some of the diseases I may be at risk for. | | | | | | |
| The results will tell me what medications to take. | | | | | | |
| The results will tell me what medications to avoid. | | | | | | |
| The results will help me change my behaviors and reduce my disease risk. | | | | | | |
| The results will help me seek medical attention and reduce my disease risk. | | | | | | |
| The results will tell me for what diseases my children are at risk. | | | | | | |
| If I learn the results I can have gene therapy to change my risk. | | | | | | |
| My participation may help researchers learn more about genes and disease. | | | | | | |
| There are no benefits. | | | | | | |

There is some other benefit not listed here (please specify):________________________.

2. What do you think the risks of the OSUMC-CPMC study are? Please state how strongly you agree or disagree with each.

| | **Strongly disagree** | **Disagree** | **Neither** | **Agree** | **Strongly agree** | **Do not want to answer** |
|---|---|---|---|---|---|---|
| My results may cause me to worry. | | | | | | |
| I may not be able to get insurance if the results get out. | | | | | | |
| I could lose my job if the results get out. | | | | | | |
| I may learn that I have an increased risk for a disease that I did not want to know about. | | | | | | |
| I may learn that I have an increased risk for a disease that I can do nothing about. | | | | | | |
| There are no risks. | | | | | | |

There is some other risk not listed here (please specify):__________________________.

3. Before enrolling in the OSUMC-CPMC study, had you ever heard about personal genomics or personalized medicine? (Select one)

__ I had **never heard** of personal genomics or personalized medicine.
__ I had **heard a little** about personal genomics or personalized medicine.
__ I had **heard a lot** about personal genomics or personalized medicine.

**Previous Genetic Testing/Genetic Counseling Experience:**

| | **Yes** | **No** | **Don't know** | **Do not want to answer** |
|---|---|---|---|---|
| Before enrolling in this study, had you ever heard about genetic testing? | | | | |
| Before enrolling in this study, had you ever had genetic testing? | | | | |
| Before enrolling in this study, had you ever met with a genetic counselor? | | | | |

**Information Seeking:**

*Internet Usage*

How often do you use the Internet?
1 = Daily
2 = Weekly
3 = Monthly

*Confidence in Information Seeking Capabilities:*

1. On a scale from 1 to 5, where 1 is Strongly Disagree and 5 is Strongly Agree, to what extent do you agree or disagree with each of the following statements:

| | **Strongly disagree 1** | **Disagree 2** | **Neither 3** | **Agree 4** | **Strongly agree 5** | **Do not want to answer** |
|---|---|---|---|---|---|---|
| You are confident in your ability to understand most health-related information. | | | | | | |
| It is easy for you to ask doctors, or other health care providers, questions. | | | | | | |
| If you don't understand something, it is easy for you to ask for help. | | | | | | |

2. Now think about the area of genetics and health. On a scale from 1 to 5, where 1 is Strongly Disagree and 5 is Strongly Agree, to what extent do you agree or disagree with each of the following statements:

| | **Strongly disagree 1** | **Disagree 2** | **Neither 3** | **Agree 4** | **Strongly agree 5** | **Do not want to answer** |
|---|---|---|---|---|---|---|
| You are confident in your ability to understand information about genetics. | | | | | | |
| It would be easy for you to get information about genetics if you wanted to. | | | | | | |
| You would be able to understand information about how genes can affect your health. | | | | | | |
| You have a good idea about how genetics may influence risk for disease generally. | | | | | | |
| You have a good idea about how your own genetic make-up might affect your risk for disease. | | | | | | |
| You would be able to explain to others how genes affect health. | | | | | | |

**Health Information Seeking:**

People get health information from many sources. The next questions are about where you get health information.

1. In the past 30 days, how often would you say you have looked for information about ways to stay healthy or to feel better?

1 = Daily
2 = Weekly
3 = Less than weekly
4 = Never

2. From which of the following sources do you currently get information about health?

| | **Yes** | **No** |
|---|---|---|
| Email or the Internet | | |
| Watching television or listening to the radio | | |
| From friends or family | | |
| Reading a book, magazine, the newspaper or other publication | | |
| Talking to a doctor or other health care provider | | |

3. What is your main source of health information from those above or any others?

☐ Email or the Internet
☐ Watching television or listening to the radio
☐ From friends or family
☐ Reading a book, magazine, the newspaper, or other publication
☐ Talking to a doctor or other health care provider
☐ Other, please specify______________________

**Adult Measures of Behavior Change:**

The next questions ask about your personal health habits.

1. Do you have any health habits that you think you should be trying to improve?

Yes ☐
No ☐

**[If no, skip the following two questions.]**

2. What are the health habits you would like to improve?

☐ Diet
☐ Exercise
☐ Reduce sun exposure
☐ Quit smoking
☐ Reduce alcohol consumption
☐ Multivitamin
☐ Other, specify_______________________

3. [Modified from a 7 point scale to 5 point scale] On a scale from 1 to 5, where 1 is Not at all confident and 5 is Very confident, how confident are you that you can make any of these changes in the next 6 months?

| Not at all Confident 1 | Somewhat Confident 2 | Neutral 3 | Confident 4 | Very Confident 5 |
|---|---|---|---|---|

**Supplemental File 2: Follow-up Survey Instrument**

**Perceived Likelihood of Disease:**

1. On a scale of 1 to 5, where 1 is certain NOT to happen and 5 is certain TO happen, what do you think is your chance of developing each of the following diseases in your lifetime?

| | **Certain NOT to Happen 1** | **2** | **3** | **4** | **Certain TO Happen 5** | **Not applicable** | **Do not want to answer** |
|---|---|---|---|---|---|---|---|
| Coronary artery disease | | | | | | | |
| Type 2 diabetes | | | | | | | |
| Iron Overload/ Hemochromatosis | | | | | | | |
| Prostate cancer | | | | | | For female participants | |
| Melanoma | | | | | | | |
| Age-related macular degeneration | | | | | | | |
| Lupus | | | | | | | |
| Type 1 diabetes | | | | | | | |
| Colorectal cancer | | | | | | | |
| Inflammatory bowel disease | | | | | | | |

**Risk Perception Accuracy:**

1. Do you have an increased risk for any of the following conditions due to a *CPMC genetic risk variant*?

| | **Yes** | **No** | **Don't know** | **Not applicable** | **Do not want to answer** |
|---|---|---|---|---|---|
| Coronary artery disease | | | | | |
| Type 2 diabetes | | | | | |
| Iron Overload/ Hemochromatosis | | | | | |
| Prostate cancer | | | | For female participants | |
| Melanoma | | | | | |
| Age-related macular degeneration | | | | | |
| Lupus | | | | | |
| Type 1 diabetes | | | | | |
| Colorectal cancer | | | | | |
| Inflammatory bowel disease | | | | | |

2. Do you have an increased risk for any of the following conditions due to your *family history*?

| | **Yes** | **No** | **Don't know** | **Not applicable** | **Do not want to answer** |
|---|---|---|---|---|---|
| Coronary artery disease | | | | | |
| Type 2 diabetes | | | | | |
| Iron Overload/ Hemochromatosis | | | | | |
| Prostate cancer | | | | For female participants | |
| Melanoma | | | | | |
| Age-related macular degeneration | | | | | |
| Lupus | | | | | |
| Type 1 diabetes | | | | | |
| Colorectal cancer | | | | | |
| Inflammatory bowel disease | | | | | |

3. Do you have an increased risk for any of the following conditions due to your *environmental risk* (for example, smoking, poor diet, high Body Mass Index (BMI))?

| | **Yes** | **No** | **Don't know** | **Not applicable** | **Do not want to answer** |
|---|---|---|---|---|---|
| Coronary artery disease | | | | | |
| Type 2 diabetes | | | | | |
| Iron Overload/ Hemochromatosis | | | | | |
| Prostate cancer | | | | For female participants | |
| Melanoma | | | | | |
| Age-related macular degeneration | | | | | |
| Lupus | | | | | |
| Type 1 diabetes | | | | | |
| Colorectal cancer | | | | | |
| Inflammatory bowel disease | | | | | |

4. On a scale from 1 to 5, where 1 is Not at all and 5 is Completely, how much do you think having a *genetic risk variant* determines whether or not a person will develop each of the following conditions?

| | **1 = Not at all** | **2** | **3** | **4** | **5 = Completely** | **Do not want to answer** |
|---|---|---|---|---|---|---|
| Coronary artery disease | | | | | | |
| Type 2 diabetes | | | | | | |
| Iron Overload/ Hemochromatosis | | | | | | |
| Prostate cancer | | | | | | |
| Melanoma | | | | | | |
| Age-related macular degeneration | | | | | | |
| Lupus | | | | | | |
| Type 1 diabetes | | | | | | |
| Colorectal cancer | | | | | | |
| Inflammatory bowel disease | | | | | | |

5. On a scale from 1 to 5, where 1 is Not at all and 5 is Completely, how much do you think *family history* determines whether or not a person will develop each of the following conditions?

| | **1 = Not at all** | **2** | **3** | **4** | **5 = Completely** | **Do not want to answer** |
|---|---|---|---|---|---|---|
| Coronary artery disease | | | | | | |
| Type 2 diabetes | | | | | | |
| Iron Overload/ Hemochromatosis | | | | | | |
| Prostate cancer | | | | | | |
| Melanoma | | | | | | |
| Age-related macular degeneration | | | | | | |
| Lupus | | | | | | |
| Type 1 diabetes | | | | | | |
| Colorectal cancer | | | | | | |
| Inflammatory bowel disease | | | | | | |

6. On a scale from 1 to 5 where 1 is Not at all and 5 is Completely, how much do you think *environmental risk factors* (for example, smoking, poor diet, high Body Mass Index (BMI)) determine whether or not a person will develop each of the following conditions?

| | **1 = Not at all** | **2** | **3** | **4** | **5 = Completely** | **Do not want to answer** |
|---|---|---|---|---|---|---|
| Coronary artery disease | | | | | | |
| Type 2 diabetes | | | | | | |
| Iron Overload/ Hemochromatosis | | | | | | |
| Prostate cancer | | | | | | |
| Melanoma | | | | | | |
| Age-related macular degeneration | | | | | | |
| Lupus | | | | | | |
| Type 1 diabetes | | | | | | |
| Colorectal cancer | | | | | | |
| Inflammatory bowel disease | | | | | | |

**General, Expanded, and Relative Risk Numeracy:**

*Expanded Numeracy Scale—EASY LEVEL QUESTION*

1. If the chance of getting a disease is 10%, how many people would be expected to get the disease:
A. Out of 100?
B. Out of 1000?

*General Numeracy Scale—MODERATE LEVEL QUESTION*

1. Imagine that we rolled a fair, six-sided die 1,000 times. Out of 1,000 rolls, how many times do you think the die would come up even (2, 4, or 6)?

*General Numeracy Scale—HARD LEVEL QUESTION*

1. In the ACME PUBLISHING SWEEPSTAKES, the chance of winning a car is 1 in 1,000. What percent of tickets to ACME PUBLISHING SWEEPSTAKES win a car?

*Relative Risk Numeracy Items*

1. People without a family history of coronary artery disease have a 20% risk to develop coronary artery disease. People with a family history have a relative risk of 2.0 (they are 2 times as likely to develop coronary artery disease as those without a family history). What is the risk for someone with a family history?
A. 40%
B. 20%
C. 22%
D. Don't know
E. Do not want to answer

2. If a person has a genetic variant that gives a relative risk for developing type 2 diabetes of 1.3, how likely are they to develop type 2 diabetes compared to someone with no copies of that genetic variant?
A. 1.3 times more likely
B. 30% more likely
C. 13 times more likely
D. Both A and B
E. None of the above
F. Don't know
G. Do not want to answer

**Knowledge of Genetics/Complex Disease/Genomics:**

1. Since you last completed the Ohio State University Medical Center-Coriell Personalized Medicine Collaborative (CPMC) survey, approximately how many times have you visited the Coriell Personalized Medicine Collaborative (CPMC) website?
    a. Once
    b. More than once, less than 5 times
    c. Between 5 times and 10 times
    d. More than 10 times
2. What is the average amount of time you spend on the CPMC website when you visit this site?
    a. Less than 5 min
    b. 5 min to 30 min
    c. 30 min to 1 h
    d. 1 h or more
3. Please tell us which sections of the CPMC website you have viewed since you last completed the OSUMC-CPMC survey (please check all that apply).
    a. Genetic education pages
    b. Results pages
    c. Health condition pages
    d. About the study
    e. How it works
4. Have you been exposed to genetics before enrolling in the CPMC (college-level courses, genetic or personalized medicine websites, articles or books)?
    a. Yes
    b. No
    c. Don't know
    d. Don't want to answer
5. Have you ever received genetic counseling?
    a. Yes
    b. No
    c. Don't know
    d. Don't want to answer

    If yes, please tell us how you received genetic counseling.
    a. By phone through the CPMC
    b. In person related to the OSUMC-CPMC study
    c. Other
    d. Don't know
    e. Don't want to answer

6. Compared to most people, how would you rate your knowledge of genetics?
    a. Better than most people
    b. About average
    c. Less than most people
7. When you have a health problem, where do you go to get information?
    a. Your doctor
    b. The internet
    c. Library
    d. Other

    If you answered Other in the question above, where do you get information about a health problem? ________________________
8. It is possible to see a gene with the naked eye.
    a. True
    b. False
    c. Don't Know
9. Healthy parents can have a child with a hereditary disease.
    a. True
    b. False
    c. Don't Know
10. The onset of certain diseases is due to genes, environment and lifestyle.
    a. True
    b. False
    c. Don't Know
11. The carrier of a disease gene may be completely healthy.
    a. True
    b. False
    c. Don't Know
12. All serious diseases are hereditary.
    a. True
    b. False
    c. Don't Know
13. Genes are inside cells.
    a. True
    b. False
    c. Don't Know
14. The child of a disease gene carrier is always also a carrier of the same disease.
    a. True
    b. False
    c. Don't Know

15. A gene is a piece of DNA.
    a. True
    b. False
    c. Don't Know
16. A gene is a part of a chromosome.
    a. True
    b. False
    c. Don't Know
17. All body parts have all of the same genes.
    a. True
    b. False
    c. Don't Know
18. It has been estimated that a person has about 20,000 genes.
    a. True
    b. False
    c. Don't Know
19. A person's race and ethnicity can affect how likely they are to get a disease.
    a. True
    b. False
    c. Not sure
20. Each of us has variations in our genes that make it more likely that we will get certain diseases.
    a. True
    b. False
    c. Not sure
21. A "complex disease" is a health condition brought on by many genes and lifestyle and environment.
    a. True
    b. False
    c. Not sure
22. A single nucleotide polymorphism or "SNiP" is a variation present in some individuals that stretches across in a large section of DNA.
    a. True
    b. False
    c. Not sure

Based on what you know about genetics, please indicate whether you think the following statements are true or false.

| | **True** | **False** |
|---|---|---|
| Once a genetic marker for a disorder is found in a person the disorder can be prevented or cured. | | |
| If a person has a genetic marker for a disorder, the person will always get the disorder. | | |
| Only mothers can pass on genetic disorders. | | |
| People who have a genetic marker for a disease are unhealthy. | | |
| A person's health habits can influence whether or not their genes cause disease. | | |

**Personalized Medicine Perceptions and Genomics Awareness:**

1. What do you think the benefits of the OSUMC-CPMC study are? Please state how strongly you agree or disagree with each.

| | **Strongly disagree** | **Disagree** | **Neither** | **Agree** | **Strongly agree** | **Do not want to answer** |
|---|---|---|---|---|---|---|
| The results told me what my genetic future is. | | | | | | |
| The results told me about all diseases I am at risk for. | | | | | | |
| The results told me about some of the diseases I may be at risk for. | | | | | | |
| The results told me what medications to take. | | | | | | |
| The results told me what medications to avoid. | | | | | | |
| The results helped me change my behaviors and reduce my disease risk. | | | | | | |
| The results helped me seek medical attention and reduce my disease risk. | | | | | | |
| The results told me for what diseases my children are at risk. | | | | | | |
| Learning my results means I can have gene therapy to change my risk. | | | | | | |
| My participation may help researchers learn more about genes and disease. | | | | | | |
| There are no benefits. | | | | | | |

There is some other benefit not listed here (please specify):_______________________.

2. What do you think the risks of the OSUMC-CPMC study are? Please state how strongly you agree or disagree with each.

| | **Strongly disagree** | **Disagree** | **Neither** | **Agree** | **Strongly agree** | **Do not want to answer** |
|---|---|---|---|---|---|---|
| My results have caused me to worry. | | | | | | |
| I may not be able to get insurance if the results get out. | | | | | | |
| I could lose my job if the results get out. | | | | | | |
| I have learned that I have an increased risk for a disease that I did not want to know about. | | | | | | |
| I have learned that I have an increased risk for a disease that I can do nothing about. | | | | | | |
| There are no risks. | | | | | | |

There is some other risk not listed here (please specify):__________________________.

**Information Seeking:**

*Internet Usage*

How often do you use the Internet?
1 = Daily
2 = Weekly
3 = Monthly

1. Since you have learned your OSUMC-CPMC results, what steps have you taken to learn more? Please check **all** that apply.

__ Read on web sites
__ Read books, brochures or other printed materials
__ Asked my physician
__ Asked another healthcare provider
__ Other: ___________________
__ I have not taken any steps to learn more about my CPMC results

*Confidence in Information Seeking Capabilities:*

1. On a scale from 1 to 5, where 1 is Strongly Disagree and 5 is Strongly Agree, to what extent do you agree or disagree with each of the following statements:

| | **Strongly disagree 1** | **Disagree 2** | **Neither 3** | **Agree 4** | **Strongly agree 5** | **Do not want to answer** |
|---|---|---|---|---|---|---|
| You are confident in your ability to understand most health-related information. | | | | | | |
| It is easy for you to ask doctors, or other health care providers, questions. | | | | | | |
| If you don't understand something, it is easy for you to ask for help. | | | | | | |

2. Now think about the area of genetics and health. On a scale from 1 to 5, where 1 is Strongly Disagree and 5 is Strongly Agree, to what extent do you agree or disagree with each of the following statements:

| | **Strongly disagree 1** | **Disagree 2** | **Neither 3** | **Agree 4** | **Strongly agree 5** | **Do not want to answer** |
|---|---|---|---|---|---|---|
| You are confident in your ability to understand information about genetics. | | | | | | |
| It would be easy for you to get information about genetics if you wanted to. | | | | | | |
| You would be able to understand information about how genes can affect your health. | | | | | | |
| You have a good idea about how genetics may influence risk for disease generally. | | | | | | |
| You have a good idea about how your own genetic make-up might affect your risk for disease. | | | | | | |
| You would be able to explain to others how genes affect health. | | | | | | |

**Health Information Seeking**

People get health information from many sources. The next questions are about where you get health information.

1. In the past 30 days, how often would you say you have looked for information about ways to stay healthy or to feel better?

   1 = Daily
   2 = Weekly
   3 = Less than weekly
   4 = Never

2. From which of the following sources do you currently get information about health?

| | **Yes** | **No** |
|---|---|---|
| Email or the Internet | | |
| Watching television or listening to the radio | | |
| From friends or family | | |
| Reading a book, magazine, the newspaper, or other publication | | |
| Talking to a doctor or other health care provider | | |

3. What is your main source of health information from those above or any others?

   ☐ Email or the Internet
   ☐ Watching television or listening to the radio
   ☐ From friends or family
   ☐ Reading a book, magazine, the newspaper or other publication
   ☐ Talking to a doctor or other health care provider
   ☐ Other, please specify______________________

**Acceptability of Test Result Information:**

On a scale from 1 to 5, to what extent do you consider the information about your genetic variant results from the OSU-Coriell PMC study to be:

| | | | | | | |
|---|---|---|---|---|---|---|
| Believable | 1 | 2 | 3 | 4 | 5 | Unbelievable |
| Trustworthy | 1 | 2 | 3 | 4 | 5 | Not trustworthy |
| Easy to understand | 1 | 2 | 3 | 4 | 5 | Hard to Understand |
| Relevant to you | 1 | 2 | 3 | 4 | 5 | Not relevant to you |
| Important to you | 1 | 2 | 3 | 4 | 5 | Not important to you |

**Family Communication:**

1. Have you told any family members about your CPMC results?

    Yes
    Not yet, but I plan to tell family members
    No, and I do NOT plan to tell any family members
    I have not decided if I will tell any of my family members yet

2. If yes/not yet, which family members have you told or do you plan to tell about your CPMC results? Please check all that apply.

    Spouse
    Mother
    Father
    Brother
    Sister
    Half brother
    Half sister
    Son
    Daughter
    Other_________________

3. If yes/not yet, why did you choose to tell or plan to tell your family members about your CPMC results? Please check all that apply.

    I think that my CPMC results are relevant to my family members' health care
    I think that my family members wanted to know
    There is a history of a certain disease in the family
    I wanted to encourage my family members to learn more about their risk
    Other ___________________________________________

4. If no, why did you choose NOT to tell your family members? Please check all that apply.

    I want to keep my CPMC results private
    I did not think my family members would want to know
    I did not think my CPMC results were relevant to my family members' health care
    I did not think my family members would approve of genetic testing
    Other_____________________________________________

**Utility of Information:**

On a scale from 1 to 5, where 1 is Strongly Disagree and 5 is Strongly Agree, to what extent do you agree or disagree with each of the following statements:

| | **Strongly disagree 1** | **Disagree 2** | **Neither 3** | **Agree 4** | **Strongly agree 5** | **Do not want to answer** |
|---|---|---|---|---|---|---|
| I feel that the information I learned through participation in the OSUMC-CPMC study is important to my health. | | | | | | |
| I feel that the information I learned through participation in the OSUMC-CPMC study will help guide my disease prevention efforts (such as getting regular exercise, making changes to my diet, and/or stopping smoking.) | | | | | | |
| I feel that my family members should have genetic testing for the CPMC genetic risk variants I have. | | | | | | |

**Adult Measures of Behavior Change**

The next questions ask about your personal health habits.

1. Do you have any health habits that you think you should be trying to improve?

   Yes ☐
   No ☐

**[If no, skip the following two questions.]**

2. What are the health habits you would like to improve?

   ☐ Diet
   ☐ Exercise
   ☐ Reduce sun exposure
   ☐ Quit smoking
   ☐ Reduce alcohol consumption
   ☐ Multivitamin
   ☐ Other, specify________________________

3. [Modified from a 7 point scale to 5 point scale] On a scale from 1 to 5, where 1 is Not at all confident and 5 is Very confident, how confident are you that you can make any of these changes in the next 6 months?

| Not at all Confident 1 | Somewhat Confident 2 | Neutral 3 | Confident 4 | Very Confident 5 |
|---|---|---|---|---|

**Supplementary Genetic/Genomic Counseling Questions:**

On a scale from 1 to 5 where 1 is Strongly Disagree and 5 is Strongly Agree, to what extent do you agree or disagree with each of the following statements:

| | **Strongly disagree 1** | **Disagree 2** | **Neutral 3** | **Agree 4** | **Strongly agree 5** | **Do not want to answer** |
|---|---|---|---|---|---|---|
| I know what to do with my results. | | | | | | |
| All individuals should meet with a genetic counselor when receiving this type of disease risk information. | | | | | | |

**Satisfaction with Genetic Counseling:**

*Genetic Counseling Satisfaction Scale*

Possible scores range from 1–5 (1 = Strongly disagree, 2 = Disagree somewhat, 3 = Uncertain, 4 = Agree somewhat, 5 = Agree strongly)

| | **Strongly disagree 1** | **Disagree somewhat 2** | **Uncertain 3** | **Agree somewhat 4** | **Agree strongly 5** | **Do not want to answer** |
|---|---|---|---|---|---|---|
| I felt better about my health after meeting with my genetic counselor. | | | | | | |
| The genetic counseling session was about the right length of time I needed. | | | | | | |
| The genetic counseling session was valuable to me. | | | | | | |
| The genetic counselor gave me information I needed. | | | | | | |

[OPTIONAL]
Please provide us with feedback about your in-person OSUMC genomic counseling appointment (*i.e.*, what you liked and/or disliked about your experience).
